# Tuberculosis Coinciding With Epidermal Growth Factor Receptor (EGFR)-Mutant Lung Adenocarcinoma: A Diagnostic Conundrum

**DOI:** 10.7759/cureus.93127

**Published:** 2025-09-24

**Authors:** Nithiyanandan Ravi

**Affiliations:** 1 Pulmonary Medicine, Apollo Hospitals, Chennai, IND

**Keywords:** adenocarcinoma lung, clinical presentation, coexistence, treatment, tuberculosis

## Abstract

Tuberculosis (TB) and lung carcinoma can coexist, creating diagnostic and therapeutic difficulties leading to poor patient outcomes, especially in a TB-endemic country like India. Here, we report an interesting case of a female patient in her 40s who presented with shortness of breath and a weight loss of 15 kg over 10 months. Her imaging findings include a right-sided pleural effusion, right parahilar lesion, and mediastinal lymphadenopathy. Medical thoracoscopy was done, and the pleural biopsy was positive for GeneXpert MTB. She responded initially to anti-TB therapy. However, she developed a new lytic lesion over the right proximal shoulder and a progression of her initial radiological findings. She was evaluated further, which revealed EGFR (epidermal growth factor receptor)-mutated lung adenocarcinoma. Hence, a diagnosis of coexistent TB and lung adenocarcinoma was made. She was lost to follow-up, and a telephonic call revealed she had died two years later.

## Introduction

Lung cancer is the most common cancer worldwide and the leading cause of cancer-related deaths worldwide [1]. Tuberculosis (TB) continues to contribute significantly to global morbidity and mortality, with particularly high incidence and death rates in TB high-burden countries, with an estimated 10.8 million new TB cases and 1.25 million TB deaths globally in 2023 [2,3]. EGFR (epidermal growth factor receptor) gene mutations are the most common targetable driver mutations seen in up to 32.3% of non-small-cell lung cancer (NSCLC) patients, and targeted therapies that improve survival highlight the need for early molecular diagnosis [4,5]. TB and malignancy may coexist in about 2% of cases, often causing discordance between radiological, histopathological, and microbiological findings, and the coexistence of TB with EGFR-mutated lung adenocarcinoma has been documented in a few case reports and case series [6-8]. Such coexistence can pose a significant diagnostic challenge due to overlapping clinical and radiological features, compounded by the high index of suspicion for TB in TB high-burden countries [2,9]. We present a case that was initially treated as TB based on microbiological evidence but, due to atypical radiological findings, was later diagnosed as coexistent TB and EGFR-mutated lung adenocarcinoma on re-evaluation, underscoring the need for biopsy of all suspicious sites in TB patients with unusual radiological presentations to rule out dual pathology.

## Case presentation

**Figure 1:**
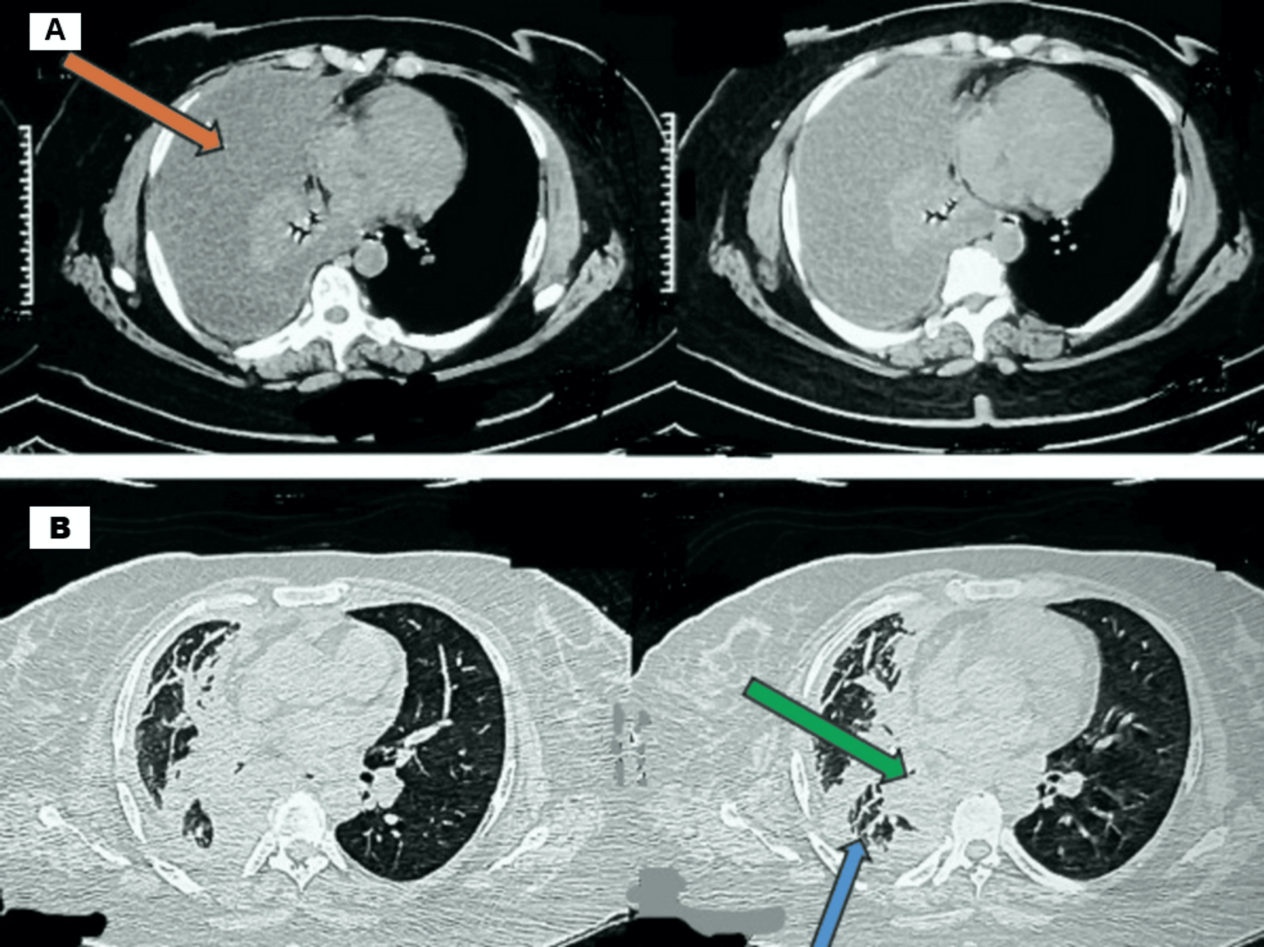
Baseline CT chest of the patient CT chest showing right-sided moderate pleural effusion with pleural thickening (orange arrow), right parahilar lesion (green arrow), and right lower lobe nodular septal thickening (blue arrow) (A) Mediastinal window image; (B) parenchymal window image CT: computed tomography

A woman in her 40s with no prior comorbidities presented with progressive weight loss of 15 kg over 10 months and shortness of breath for one month. On examination, she was tachypneic (respiratory rate (RR) 30/minute) with decreased breath sounds on the right side of the chest; other vital signs were normal. Initial chest X-ray revealed a large right-sided pleural effusion. She underwent ultrasound-guided thoracocentesis and chest tube placement, draining 500 mL of yellowish pleural fluid. Laboratory analysis of the fluid showed a total leukocyte count of 2,420/mm³ (85% lymphocytes), glucose 115 mg/dL, protein 4.88 g/dL, lactate dehydrogenase (LDH) 245 IU/L, and adenosine deaminase (ADA) 29.16 IU/mL. Microbiological studies including culture, acid-fast bacilli (AFB) smear, GeneXpert MTB, and cytology were inconclusive. Contrast-enhanced computed tomography (CECT) chest demonstrated moderate right pleural effusion with pleural thickening, a right parahilar lesion, right lower lobe nodular septal thickening, and mediastinal lymphadenopathy (Figures 1A, 1B).

**Figure 2:**
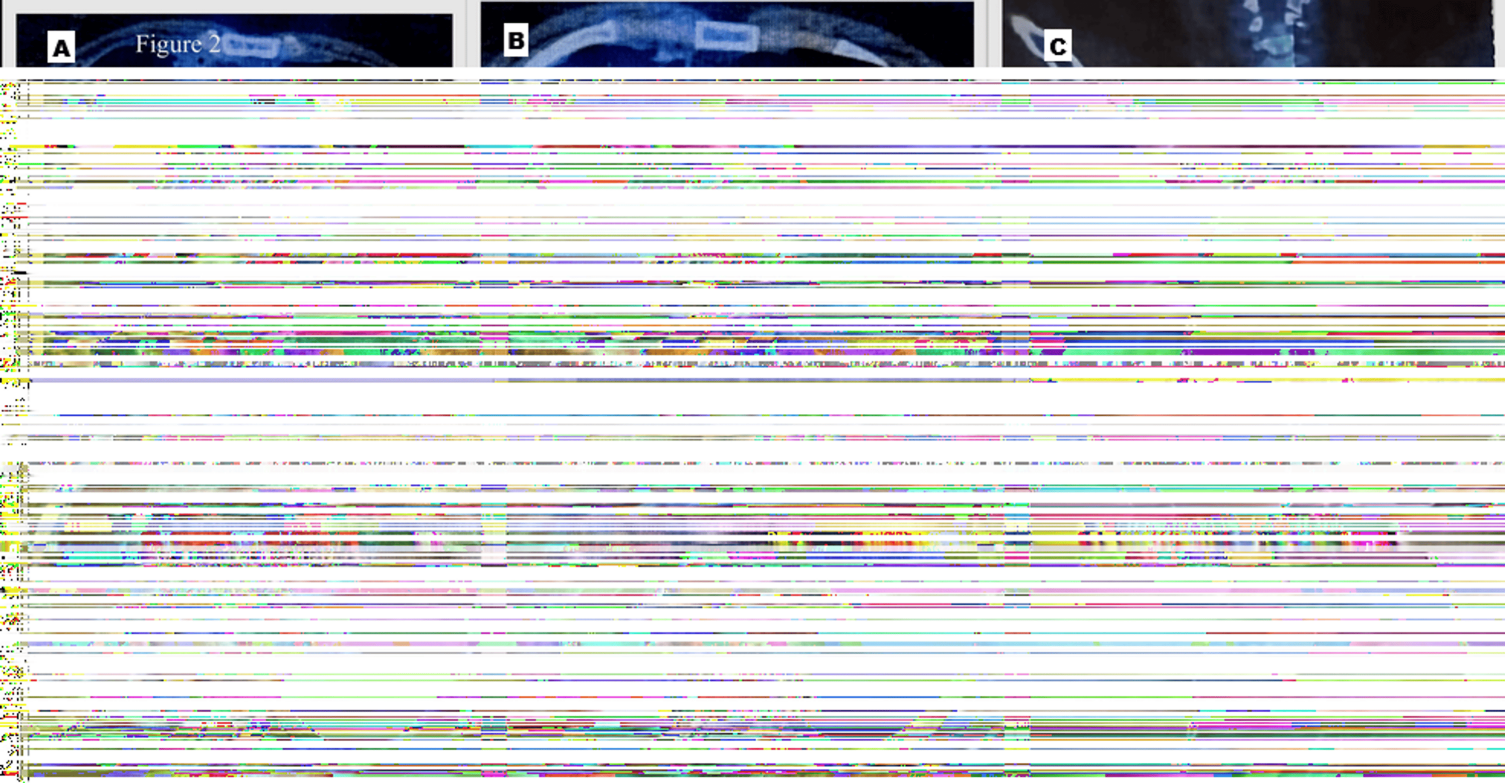
PET scan findings Baseline: (A) right parahilar lesion (23 x 17 mm, SUVmax 15.4); (B) pre-tracheal (4R) lymph node enlargement (29 x 22 mm, SUVmax 15.3); (C) normal humerus After two months of ATT: (D) right parahilar lesion (26 x 26 mm, SUVmax 15.6); (E) mediastinal lymph node enlargement (31 x 29 mm, SUVmax 16.9); (F) lytic lesion over the proximal right humerus (SUVmax 6.9) ATT: anti-tuberculous therapy; PET: positron emission tomography

**Figure 3:**
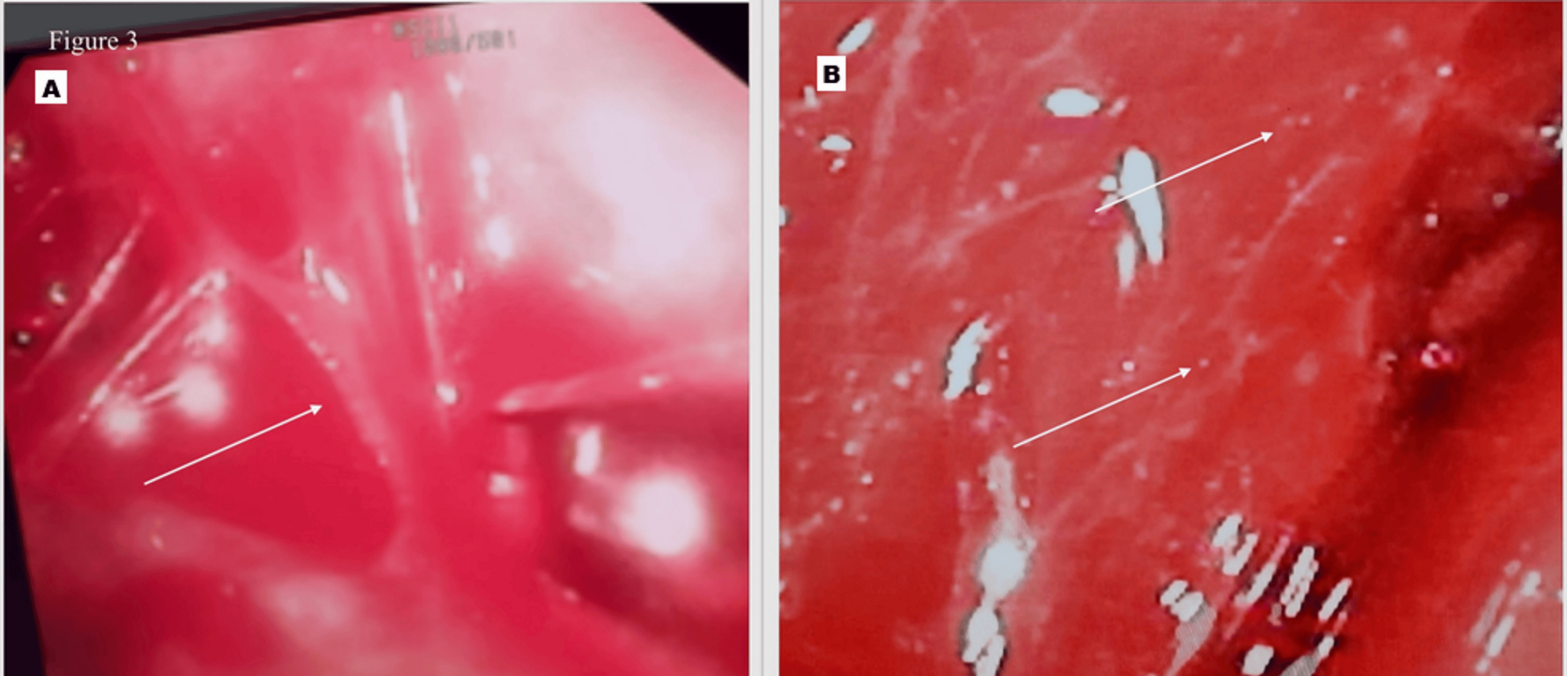
Medical thoracoscopy images of the patient (A) Extensive pleural adhesions (shown by a white arrow); (B) pleural nodules giving a sago-grain appearance (shown by white arrows)

Whole-body positron emission tomography (PET) scan confirmed pleural thickening (SUVmax 3.8), a right parahilar lesion (23 × 17 mm, SUVmax 15.4), lower lobe nodules (largest 12 × 12 mm, SUVmax 2.4), and mediastinal lymphadenopathy (29 × 22 mm, SUVmax 15.3) (Figure 2), raising clinical suspicion for malignancy. Pleural fluid AFB culture was negative, and medical thoracoscopy revealed multiple small pleural nodules resembling sago grains (Figure 3). Biopsy showed chronic inflammation with lymphocyte infiltration and no granulomas, and GeneXpert MTB was highly detected (rifampicin resistance not detected).

**Figure 4:**
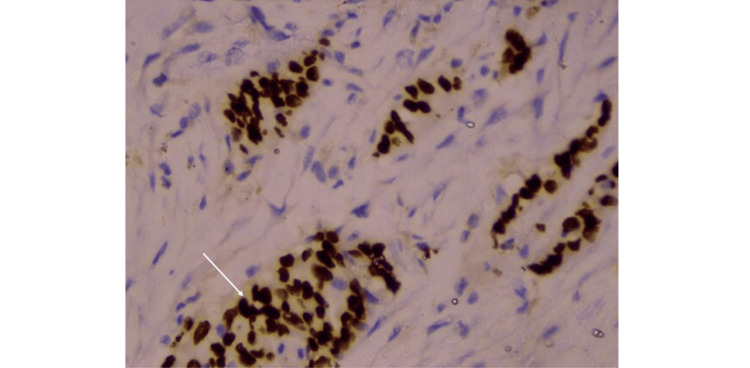
Core needle biopsy of EBUS-TBNA specimen from right parahilar lesion EBUS-TBNA from right parahilar lesion showing strong nuclear positivity for TTF-1 by immunohistochemistry (white arrow) in a background of atypical cells, confirming the diagnosis of primary lung adenocarcinoma EBUS-TBNA: endobronchial ultrasound-guided transbronchial needle aspiration; TTF-1: thyroid transcription factor 1

She was started on weight-based anti-tuberculous therapy (ATT) (2HRZE + 4HRE); after two months, she showed stabilization of weight and improvement in dyspnea, and follow-up chest X-ray demonstrated stability of the pleural effusion and right parahilar lesion. During follow-up, she developed right shoulder pain with local tenderness, prompting repeat PET imaging after three months of anti-TB treatment, which showed increased right pleural effusion and thickening (SUVmax 9.7), enlargement of the right parahilar lesion (26 × 26 mm, SUVmax 15.6), mediastinal lymphadenopathy (31 × 29 mm, SUVmax 16.9), and a new lytic lesion in the right proximal humerus (SUVmax 6.9) (Figure 2). These findings raised suspicion for alternate or coexistent pathology, and endobronchial ultrasound-guided transbronchial needle aspiration (EBUS-TBNA) from the right parahilar lesion and mediastinal lymph node revealed atypical cells and immunohistochemistry (IHC) positivity for thyroid transcription factor 1 (TTF-1) consistent with metastatic lung adenocarcinoma (Figure 4), while microbiology remained negative. Next-generation sequencing identified an EGFR exon 21 missense mutation, confirming a diagnosis of concomitant pleural TB with EGFR-mutated metastatic lung adenocarcinoma (TNM 8 stage T3N2M1b: IVA). She was counseled regarding her diagnosis and treatment options but was lost to follow-up, with a telephonic update revealing she had died two years after diagnosis. Table 1 summarizes the clinical course and key events in the management of the index patient.

**Table 1:** Timeline of clinical events and management of the index patient CECT: contrast-enhanced computed tomography; PET: positron emission tomography; ADA: adenosine deaminase; AFB: acid-fast bacilli; SUVmax: maximum standardized uptake value; ATT: anti-tuberculous therapy; EBUS-TBNA: endobronchial ultrasound-guided transbronchial needle aspiration; IHC: immunohistochemistry; TTF-1: thyroid transcription factor 1; NGS: next-generation sequencing; EGFR: epidermal growth factor receptor; CT: computed tomography

Timeline	Event/symptom	Key investigations	Findings	Interpretation and intervention
Presentation	Weight loss 15 kg over 10 months, dyspnea, large right pleural effusion	Pleural fluid analysis	Lymphocytic, exudative, low ADA, GeneXpert MTB, AFB smear, and culture-negative	Inconclusive and planned for further evaluation
		CECT chest and PET-CT (Figures *1A*, *1B*, 2)	Pleural thickening, right parahilar lesion, nodular septal thickening, mediastinal lymphadenopathy (SUVmax 3.8-15.4)	Suspicion of malignancy
Thoracoscopy	Pleural biopsy	Histopathology and GeneXpert MTB (Figure *3*)	Chronic inflammation with no granulomas GeneXpert MTB: high, rifampicin sensitive	ATT started; patient counseled regarding the need for close follow-up citing atypical radiological findings
2 months post ATT	Follow-up	Clinical and X-ray chest	Weight stabilized, dyspnea improved, pleural effusion and right parahilar lesion stable	Continued ATT
3 months post ATT	Right shoulder pain	PET-CT (Figure *2*)	New-onset lytic humeral lesion; increased pleural effusion & lesion size (SUVmax 9.7–16.9)	Re-evaluation for dual pathology
	EBUS-TBNA and IHC	Core biopsy (Figure *4*)	Atypical cells, TTF-1 positive → metastatic lung adenocarcinoma, AFB smear: negative/GeneXpert: negative	NGS panel
	NGS panel	Molecular testing	EGFR exon 21 mutation	Final diagnosis: pleural TB + EGFR-mutated metastatic adenocarcinoma
2 years after diagnosis	Outcome assessment	Telephonic follow-up	Treatment details not known	Deceased two years after diagnosis

## Discussion

This case underscores the diagnostic and therapeutic challenges of coexistent TB and EGFR-mutated lung adenocarcinoma. The patient presented with several red flag signs like massive pleural effusion, low ADA levels, nodular pleural thickening, absence of fever, and significant functional limitation. Although the thoracoscopic pleural biopsy was GeneXpert MTB-positive, the absence of granulomas and negative AFB smear and culture raised concerns about diagnostic accuracy. False-positive GeneXpert results, though rare, are described in the literature-often in the setting of “low detected” values from non-viable bacilli or contamination [10]. In our case, the “high detected” result with no prior TB history made this explanation less likely. Discordance between GeneXpert and pleural culture has been reported, reflecting technical limitations and sampling variability [11]. With GeneXpert showing pooled sensitivity and specificity of ~85.5% and 97.2%, respectively, compared to the higher sensitivity (92.7%) and near-perfect specificity (100%) of pleural biopsy culture, initiation of ATT with close monitoring was considered appropriate while keeping the possibility of dual pathology in mind [12].

The patient initially improved clinically, but subsequent development of new symptoms and progressive PET findings, including a lytic bone lesion, prompted re-evaluation. EBUS-TBNA confirmed metastatic EGFR exon 21-mutated adenocarcinoma coexisting with pleural TB. Such diagnostic complexity arises from several factors: pleural TB is a paucibacillary disease where microbiological confirmation is difficult; pleural biopsy may miss focal granulomas; both TB and malignancy can produce effusion, adenopathy, and systemic symptoms; and in TB-endemic regions, high background prevalence biases clinicians toward TB, sometimes delaying recognition of cancer [2,13,14]. Multi-site sampling and early use of modalities such as EBUS-TBNA are critical to avoid misclassification. The coexistence of TB and lung cancer has biological plausibility. Chronic TB inflammation and scarring can predispose to “scar carcinoma,” mycobacterial cell wall components may induce oxidative DNA damage, and lung carcinoma itself can reactivate latent TB; additionally, cancer treatment can suppress immunity and predispose to TB [15-17]. In the index case, the first three mechanisms appear most plausible. Previous case series and case reports suggest an increased occurrence of EGFR-mutated lung cancer in TB patients especially among women and never-smokers [7,18,19]. A single-center prospective observational study conducted in India showed a high prevalence of 16% for TB and NSCLC coexistence. Adenocarcinoma (36%) seems to be the most common cancer sub-type associated with TB, followed by squamous cell carcinoma (34%). Also, survival rates of coexisting pathologies were lower compared to either pathology occurring alone [8]. The treatment plan for coexistent TB and EGFR-mutated lung adenocarcinoma is not clear, considering the drug interactions between rifampicin and targeted therapies. Rifampicin reduces the plasma concentration of TKIs (tyrosine kinase inhibitors), and hence, it is advisable to increase the dosage of TKIs and monitor the levels of TKIs in the plasma. However, if measurement of the plasma concentration of TKI is not possible, it is advisable to start ATT without rifampicin and prolong the duration of TB treatment to nine months [20]. Our index case was on full-course ATT including rifampicin at the time of her last follow-up, and further details of her cancer treatment were not known.

## Conclusions

This case underscores the complex diagnostic and therapeutic challenges posed by the coexistence of TB and EGFR-mutated lung adenocarcinoma. Pleural TB being paucibacillary, overlapping radiological and clinical features, and sampling limitations can obscure or delay the recognition of malignancy, particularly in TB-endemic regions where clinicians may be biased toward TB. A high index of suspicion, early use of multi-site sampling and EBUS-TBNA, and continuous follow-up are critical for timely diagnosis. Management further requires careful consideration of drug-drug interactions between ATT and TKIs, highlighting the need for individualized treatment strategies in such dual pathology.
